# Balancing Purification and Ultrastructure of Naturally Derived Bone Blocks for Bone Regeneration: Report of the Purification Effort of Two Bone Blocks

**DOI:** 10.3390/ma12193234

**Published:** 2019-10-02

**Authors:** Mike Barbeck, Ole Jung, Xin Xiong, Rumen Krastev, Tadas Korzinskas, Stevo Najman, Milena Radenković, Nils Wegner, Marina Knyazeva, Frank Walther

**Affiliations:** 1 Department of Oral and Maxillofacial Surgery, Working Group Biomaterials/Surfaces, University Hospital Hamburg-Eppendorf, Hamburg 20246, Germany; ole.tiberius.jung@googlemail@com (O.J.); tadaskorzinskas@yahoo.de (T.K.); 2 BerlinAnalytix GmbH, Berlin 12109, Germany; 3 NMI, Natural and Medical Sciences Institute at the University of Tübingen, Reutlingen 72770, Germany; xin.xiong@nmi.de; 4 Faculty of Applied Chemistry, Reutlingen University, Reutlingen 72770, Germany; rumen.krastev@reutlingen-university.de; 5 Department for Cell and Tissue Engineering and Department of Biology and Human Genetics, Faculty of Medicine, University of Niš, 18100 Niš, Serbia; stevo.najman@medfak.ni.ac.rs; 6 Department for Cell and Tissue Engineering, Faculty of Medicine, University of Niš, Niš 18100, Serbia; milena.radenkovic@pmf.edu.rs; 7 Department of Materials Test Engineering (WPT), TU Dortmund University, Dortmund 44227, Germany; nils.wegner@tu-dortmund.de (N.W.); marina.knyazeva@tu-dortmund.de (M.K.); frank.walther@tu-dortmund.de (F.W.)

**Keywords:** bone block, allogeneic, xenogeneic, purification, bone regeneration, dentistry

## Abstract

The present publication reports the purification effort of two natural bone blocks, that is, an allogeneic bone block (maxgraft^®^, botiss biomaterials GmbH, Zossen, Germany) and a xenogeneic block (SMARTBONE^®^, IBI S.A., Mezzovico-Vira, Switzerland) in addition to previously published results based on histology. Furthermore, specialized scanning electron microscopy (SEM) and in vitro analyses (XTT, BrdU, LDH) for testing of the cytocompatibility based on ISO 10993-5/-12 have been conducted. The microscopic analyses showed that both bone blocks possess a trabecular structure with a lamellar subarrangement. In the case of the xenogeneic bone block, only minor remnants of collagenous structures were found, while in contrast high amounts of collagen were found associated with the allogeneic bone matrix. Furthermore, only island-like remnants of the polymer coating in case of the xenogeneic bone substitute seemed to be detectable. Finally, no remaining cells or cellular remnants were found in both bone blocks. The in vitro analyses showed that both bone blocks are biocompatible. Altogether, the purification level of both bone blocks seems to be favorable for bone tissue regeneration without the risk for inflammatory responses or graft rejection. Moreover, the analysis of the maxgraft^®^ bone block showed that the underlying purification process allows for preserving not only the calcified bone matrix but also high amounts of the intertrabecular collagen matrix.

## 1. Introduction

In dentistry and many surgical disciplines, different allogeneic and xenogeneic bone substitutes are available [1,2]. This material class, including the so-called naturally derived bone substitutes, is expected to exhibit favorable regenerative properties based on the “natural” chemical composition and ultrastructure of bone tissue, which is supposed to be comparable to autologous bone transplants [3]. However, a prerequisite for their safe and effective clinical application is the purification of the precursor tissue from all immunologically effective components such as the different cell types or proteins as well as possibly existing pathogens [4,5]. In this context, the overall aim of every purification technology is the preservation of the ultrastructure of the bone matrix in combination with collagen from the intertrabecular tissue to optimally support the process of bone regeneration and associated healing factors such as the implant bed vascularization [3,5,6]. 

Interestingly, a variety of purification techniques with different physical and/or chemical methods is applied in case of the different commercially available bone blocks that should lead to the desired final naturally derived biomaterial [3,7]. In this context, most of the material manufacturers have introduced their own purification method and all of these methods are stated to fulfill the relevant rules, here the standards and respective norms [7]. However, a previous study, focused on the analysis of the structure of two allogeneic and three xenogeneic bone blocks to assess whether the components, which should be removed, can be identified after applying conventional histological and histochemical staining techniques, revealed wide variations between the purification efforts of the different commercially available bone blocks [7]. In this study, the bone blocks were divided into four different groups due to their respective ultrastructure of the bone matrix and organic contents (collagen, cell remnants) [7]. This classification varies from the complete purification of the natural bone substitute with loss of its lamellar structure up to full conservation of the bone matrix with its lamellar and collagenous structures. Although it is questionable if the observed remnants are still biologically active and may cause inflammatory responses up to a complete rejection of the draft, a first selection based on even such components may help clinicians to choose the right bone substitute material. 

Based on these previously reported results, the aim of the present study was the additive analysis of the ultrastructure of two other commercial available bone substitute blocks, the allogeneic block maxgraft^®^ (botiss biomaterials GmbH, Zossen, Germany) and the xenogeneic block SMARTBONE^®^ (IBI S.A., Mezzovico-Vira, Switzerland), with special respect to the microscopical analysis of the calcified bone matrix as well as the detection of other components such as collagen and possible organic remnants. Thereby, the surface and microstructure was additionally investigated by scanning electron microscopy (SEM) and a standardized cytocompatibility analysis based on ISO 10993-5/-12 as previously described [8].

## 2. Materials and Methods

### 2.1. Bone Blocks

#### 2.1.1. Maxgraft^®^ Block

Three samples of both bone blocks were histologically prepared and investigated to determine the composition accordingly to the previous published methods [7,9,10]. Special focus was on the detection of possible organic components, and thus on the control and assurance of the purification quality as previously described [7].

The maxgraft^®^ bonebuilder (botiss biomaterials GmbH, Berlin, Germany) is an allogeneic cancellous bone substitute block derived from bone of femoral heads of living human donors from Germany, Austrian and Swiss hospitals [9]. The bone blocks were prepared by the Cells + Tissuebank Austria, a certified and audited non-profit organization, which is regulated by the Austrian health ministry [11]. The purification of the bone tissue is stated to be in accordance with the respective European Directives and the Austrian Tissue Safety Act [9]. The effort of this purification process should have been “validated by independent institutes” and by the Austria Health Ministry [11].

The purification process, the “C+TBA process”, is only described in more detail at the manufacturer´s homepage [11]. It is described as a highly secure quality process, which should be in compliance with highest quality standards leading to the inactivation of viruses and bacteria [11]. Furthermore, this purification process is described to include different physical and chemical purification steps [11]. Initially, an ultrasonic-based removal of blood, cells and tissue components, which is stated to mainly have an effect on the removal of fatty tissue, is applied as a physical method. Additionally, chemical and oxidative cleaning steps using diethyl ether and ethanol with changing durations become applied to inactivate both pathogens such as viruses and bacteria and also proteins [11]. Finally, lyophilization and sterilization via gamma irradiation are applied, whereby the lyophilization is described to preserve the natural tissue structure [11]. It is stated that the final composition of the bone block includes the bone matrix and the bone tissue-specific collagen [11].

#### 2.1.2. SMARTBONE^®^ Block

The SMARTBONE^®^ block (Industrie Biomediche Insubri SA, Mezzovico-Vira, Ticino, Switzerland) is a xenogeneic bone substitute material, which is based on a bovine matrix that becomes combined with “biodegradable polymers” and “cell nutrients” [12]. However, no detailed information is given about the last-named additions. The “biodegradable polymer” is only described to be “the same as used in resorbable sutures” [12]. Interestingly, different combinations have been described in the patent application publication, while the combination with a polycaprolactone–polylactic (PLA/PCL) co-polymer and hydrolyzed gelatin has been highlighted in the document [12]. Moreover, it is stated that that the bone block includes bone tissue-specific collagen [12]. The bovine bone matrix originates from animals from New Zealand, stated as a “bovine spongiform encephalopathy (BSE)free country”, without further information about control mechanisms of the origin tissue and is only stated as “strictly monitored according to ISO13485 prescriptions“ [12,13]. Also no detailed information about the purification procedure is given on the package insert nor on the website, but after an email contact with the manufacturer the process was described as “a patented method”, which includes “bone washing and cleaning via chain-reaction and low-temperature (<50 °C) combined with undefined “chemical processes”, which “not imply calcination“. Finally, “the starting material should not be ceramic-like”, but “a natural material conserving the optimal mechanical properties of a bovine-originated mineral structure”.

### 2.2. Scanning Electron Microscopy (SEM) 

In order to characterize the surface morphology of both allogenic and xenogenic blocks, the investigation was performed by means of a scanning electron microscope (CROSSBEAM XB 550L ZEISS, Oberkochen, Germany) equipped with a charge compensator. This method allows surface examination of uncoated non-conducting materials due to gas inflow through the needle of charge compensator. The chosen parameters (accelerating voltage of 3 kV, working distance of 10–13 mm) helped to prevent surface damage due to the scanning electron beam and provided information about collagen remnants. The gas inflow was applied only for minimizing of charging effects; no images were acquired.

### 2.3. Histological Analysis

Three samples of both bone blocks were histologically processed for further microscopic ex vivo examination as described previously [7,9,10]. Briefly, all test samples were decalcified in tris-buffered 10% EDTA (Carl Roth, Karlsruhe, Germany), dehydrated in a series of increasing alcohol concentrations (50, 70, 70, 80, 96, 100 and 100%) followed by the application of xylol and paraffin embedding. Afterwards, histological sections of 3–5 μm thickness were prepared using a rotation microtome (Leica RM2255, Wetzlar, Germany). The following histochemical stainings were prepared: hematoxylin and eosin (HE), Masson–Goldner’s trichrome, Giemsa and Sirius red, respectively. Osteoclasts were determined by employing tartrate-resistant acid phosphatase (TRAP).

The histological analysis of the compositions of both bone blocks was also conducted following previously described methods [7,9,10,14,15]. Briefly, the histological slides of the bone substitutes were examined microscopically with respect to material characteristics such as the bone matrix structure and components like collagen or cells/cell remnants independently and blind by the two first authors using a light microscope Axio Scope.A1 (Carl Zeiss Microscopy GmbH, Jena, Germany). A Nikon DS-Fi1 digital camera and a DS-L2 digital sight control unit (both Nikon, Tokyo, Japan) were used in combination with the above-mentioned microscope to take the histological microphotographs.

### 2.4. Cytocompatibility Analyses

Cytocompatibility was determined based on EN ISO 10993-5/-12 regulations as described in previous publications [8,16]. In the following, the experimental setup is described in brief. Overall, *n* = 8 extracts of each test sample were processed.

#### 2.4.1. Reference Materials (Positive and Negative Controls)

As a positive control material, RM-A, a polyurethane film containing 0.1% zinc diethyldithiocarbamate (ZDEC) obtained from the Hatano Research Institute, Food and Drug Safety Center, Tokyo, Japan, was employed. Titanium grade 4 was utilized as a negative control material. All reference materials were prepared with identical surface areas as the material specimens and sterilized likewise. 

#### 2.4.2. Cell Culture

L-929 mouse fibroblasts were acquired from the European Collection of Cell Culture, ECACC (Salisbury, UK). Cells were cultured in a cell culture medium under cell culture conditions and passaged at 80% confluency. 

#### 2.4.3. Extraction

All samples were extracted for 72 h at a surface to volume ratio of 3 cm^2^/mL in a cell culture medium under cell culture conditions. The cell culture medium was incubated under comparable conditions as a blind control (not displayed in results). After removal of the specimens, the remaining extracts were centrifuged at 14,000 rpm for 10 min. The supernatants were used for the different assays that described below.

#### 2.4.4. Assay Procedure

The 96-well plates were seeded with 1 × 10^4^ L929 cells/well in 100 µL cell culture medium and incubated under cell culture conditions for 24 h before end of extraction. After delivering the cell culture medium to the waste, 100 µL extracts were given to every cell well. After an incubation interval of 24 h, BrdU and XTT assays were performed and supernatants were used for the LDH assay. 

#### 2.4.5. XTT-Assay

Cell Proliferation Kit II (XTT) (Roche Diagnostics, Mannheim, Germany) was utilized according to the manufacturer's instructions. In brief, electron-coupling reagent was mixed with XTT labeling reagent (1:50 dilution) and 50 µL of the mixture was added to the cells. Cells were incubated for a total time interval of 4 h under cell culture conditions. The absorbances of 100 µL aliquots were determined using a scanning multi-well spectrophotometer (Biorad 680, Hercules, CA, USA) with filters for 450 nm and 650 nm (reference wavelength).

#### 2.4.6. BrdU-Assay

BrdU (colorimetric) test kit (Roche Diagnostics, Mannheim, Germany) was employed according to the manufacturer's instructions. In brief, cells were fixed with FixDEnat at room temperature for 30 min after labelling with BrdU for a time interval of 2 h. Subsequently, anti-BrdU-POD antibody was used for 1 h before washing several times in washing buffer. Tetramethylbenzidine (TMB) was added for 20 min at room temperature before adding 25 µL 1 M H_2_SO_4_ stopping reaction. A scanning multi-well spectrophotometer (ELISA reader) with 450 nm and 690 nm (reference wavelength) filters was used to determine absorbances.

#### 2.4.7. LDH-Assay

An LDH-Cytotoxicity Assay Kit II (BioVision, Milpitas, CA, USA) was employed according to the instructions of the manufacturer. Accordingly, 10 µL of the extracted cell supernatants were mixed with 100 µL LDH reaction reagent at room temperature for a time interval of 30 min. Thereafter, stop solution was added and absorbances were determined by using a multi-well spectrophotometer (ELISA reader) with filters for 450 nm and 650 nm (reference wavelength).

### 2.5. Statistical Analyses 

Data were analyzed using ANOVA analysis with a post hoc Bonferroni test using GraphPad Prism 8.02 software (GraphPad Software Inc., San Diego, CA, USA). Statistical differences were specified as follows: Significant for *p*-values less than 0.05 (* *p* ≤ 0.05) and highly significant if *p*-values were less than 0.01 (** *p* ≤ 0.01). 

## 3. Results

### 3.1. Microscopic Characterization

**Figure 1:**
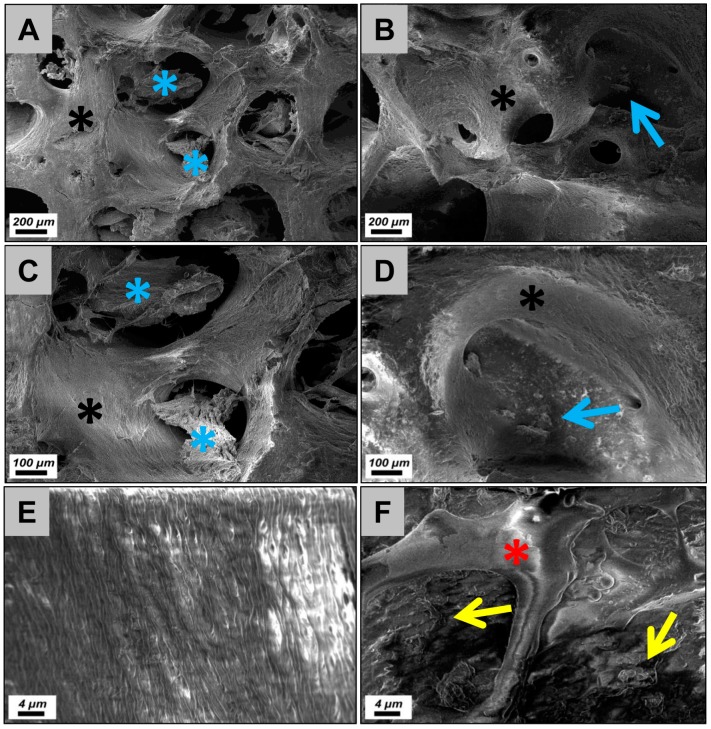
Scanning electron microscopy (SEM) images of the two bone blocks. The analysis revealed that the maxgraft^®^ block (black asterisks = calcified bone matrix) contained high amounts of intertrabecular collagen (blue asterisks) (**A** and **C**), while only some collagen spots (blue arrow) were found in case of the SMARTBONE^®^ block (**B** and **D**). Moreover, the SEM analysis showed that the maxgraft^®^ bone block exhibited a fibrillar surface texture (**E**), while island-like areas of the added polymer (red asterisks in **F**) and some loosely distributed collagen fibers were observable in case of the SMARTBONE^®^ block (**A** and **B**: 50× magnification, **C** and **D**: 100× magnification, **E** and **F**: 2000× magnification).

**Figure 2:**
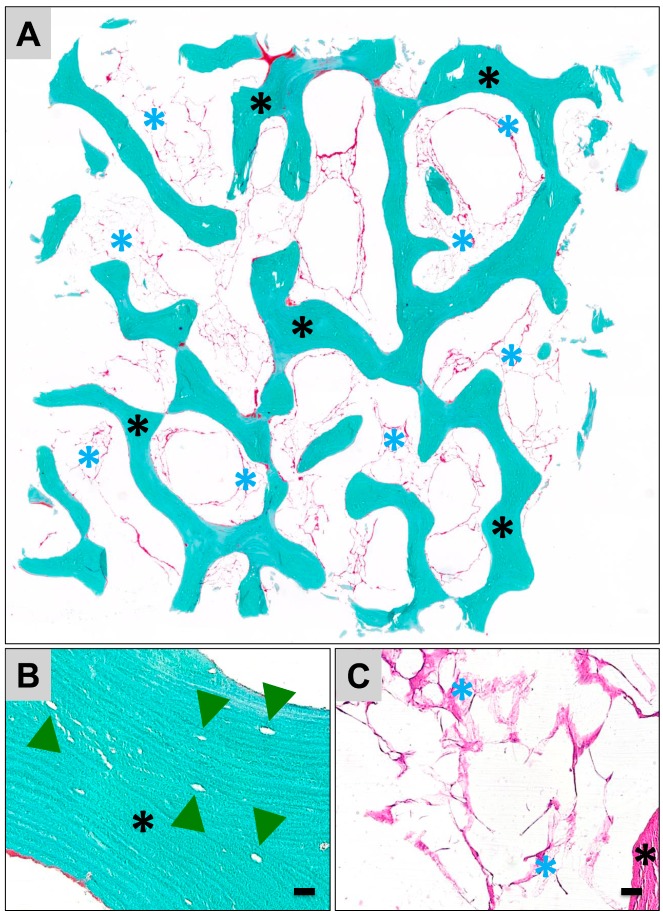
Representative histological images of the ultrastructure of the allogeneic maxgraft^®^ bone block. (**A**) Overview of the bone block that shows its trabecular structure (black asterisks = trabeculae). Within the trabecular interspaces, high amounts of collagen were overserved (blue asterisks) (Masson–Goldner staining, “total scan”, 10× magnification). (**B**) maxgraft^®^ shows a lamellar bone matrix organization. No signs of cells or cell remnants were detected within the osteocyte lacunae (green arrow heads) nor adherent to the outer surfaces of the bone matrix (Masson–Goldner staining, 40× magnification, scale bar = 10 µm). (**C**) Within the trabecular interspaces (black asterisk = trabecula) collagenous structures (blue asterisks) were observable (hematoxylin and eosin (HE)-staining, 40× magnification, scale bar = 10 µm).

**Figure 3:**
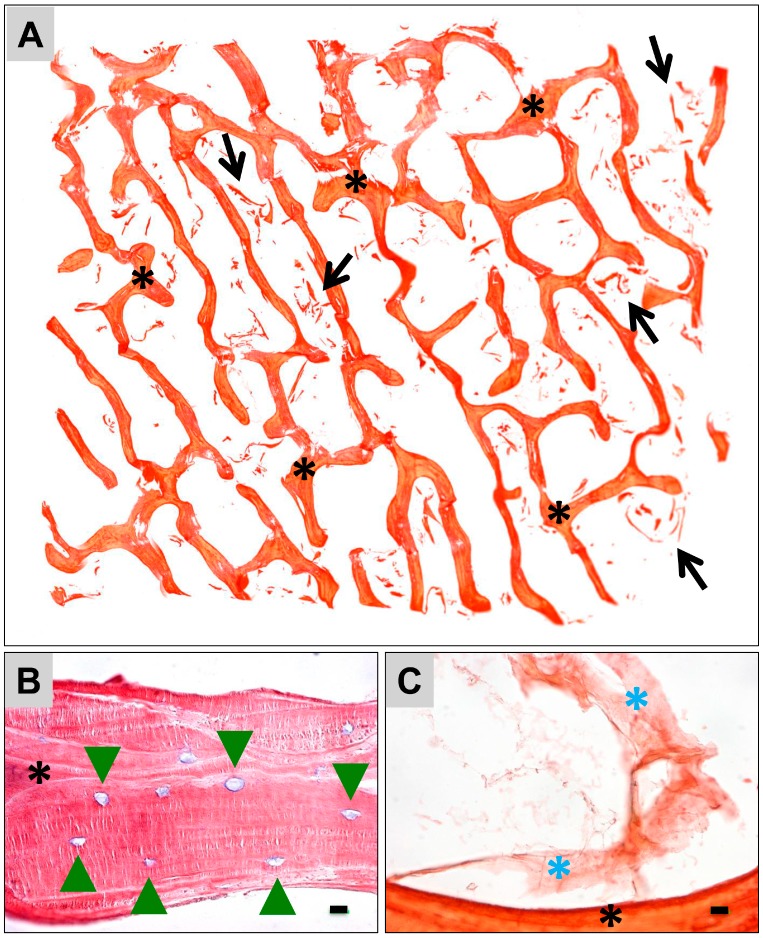
Representative histological images of the ultrastructure of the xenogeneic SMARTBONE^®^ bone block. (**A**) The bone block shows a trabecular structure (asterisks = trabeculae). Within the trabecular interspaces, high numbers of matrix fragments were overserved (arrows) (Sirius red-staining, “total scan”, 10× magnification). (**B**) SMARTBONE^®^ shows a lamellar bone matrix organization. No signs of cells or cell remnants were detected within the osteocyte lacunae (green arrow heads) nor adherent to the outer surfaces of the bone matrix (Giemsa staining, 40× magnification, scale bar = 10 µm). (**C**) Within the trabecular interspaces (black asterisk = trabecula) collagenous structures (blue asterisks) were identifiable (Sirius red staining, 60× magnification, scale bar = 1 µm).

The microscopic characterization via scanning electron microscopy (SEM) and via histology combinatorially revealed that both bone blocks possess a trabecular structure typical for the calcified bone matrix (Figure 1A–D, Figure 2A and Figure 3A). Moreover, both biomaterials exhibited a lamellar substructure, and neither cells nor cell remnants were observable within the osteocyte lacunae nor at their outer surfaces (Figure 1, Figure 2B,C and Figure 3B,C). No signs of tartrate-resistant acid phosphatase (TRAP)-positive cells were observed in case of both bone substitute materials (data not shown).

Furthermore, a few fragments of the bone matrix were observable within the trabecular interspaces in case of the allogeneic bone block (Figure 2A), while within the intertrabecular interspaces of the xenogeneic bone block high numbers of matrix fragments were observed (Figure 3A). 

Additionally, both microscopy methods showed that high amounts of the intertrabecular collagenous matrix were observable in all analyzed allogeneic bone blocks (Figure 1A,C, and Figure 2A,C). The morphology of the collagenous structures was similar to fatty tissue and connective tissue (Figure 2C). In contrast, only some loosely distributed collagen remnants were found at the surfaces of the xenogeneic bone blocks (Figure 1B,D,F and Figure 3C). The SEM analysis showed that island-like areas that seemed to originate from the added polymer were observable in case of the xenogeneic SMARTBONE^®^ block (Figure 1F), while the maxgraft^®^ bone block exhibited a fibrillar surface texture (Figure 1E).

Based on these new results, both bone blocks are comparable to the bone blocks of group 2 from the previously conducted study, which means that the level of purification as well as the preservation of the bone matrix is similar to the DIZG Human Spongiosa block [6]. However, the purification level of both bone blocks seems to be more favorable as not only the “intact” calcified bone matrix was detected but also tissue-specific collagen, especially in case of the maxgraft^®^ blocks. Thus, the former classification of bone blocks must be revised and three new groups regarding the quality standards need be added (Table 1).

### 3.2. Cytocompatibility Analysis

**Figure 4:**
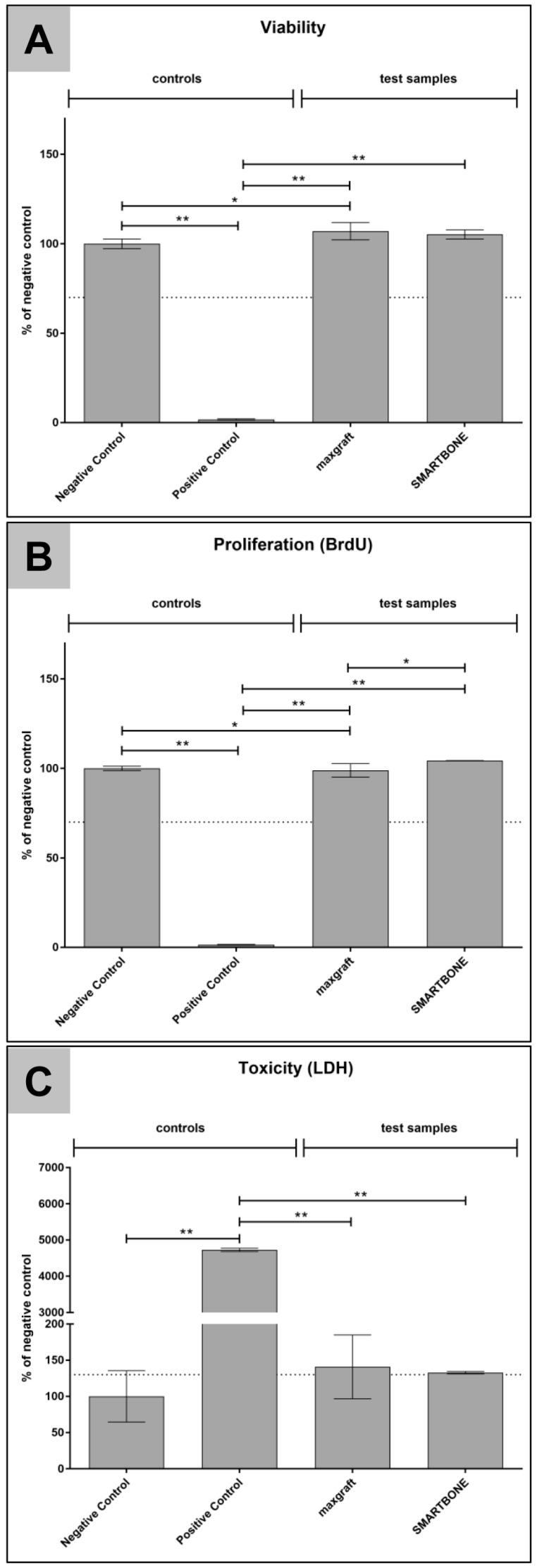
Cytocompatibility results of the different variants. (**A**) Viability measured by a BrdU assay; (**B**) proliferation measured by a Sodium 3,3′-[1(phenylamino)carbonyl]-3,4-tetrazolium]-3is(4-methoxy-6-nitro) benzene sulfonic acid hydrate (XTT)-assay; (**C**) cytotoxicity measured by a lactate dehydrogenase (LDH) assay. Values are normalized against the respective negative control. Means with error bars indicate standard deviations. The dotted line indicates thresholds which should not be exceeded (LDH) or fall below (XTT; BrdU). Significant differences are indicated as described in the results section.

According to EN ISO 10993-5:2009, values ≥ 70% relative to the negative control in the XTT and BrdU assays and values ≤ 130% of the negative control in the LDH assay display the nontoxic range (Figure 4) [8]. In the BrdU and XTT assays, the values for maxgraft^®^ and SMARTBONE^®^ were within the nontoxic range (Figure 4A,B). 

In the BrdU assay, maxgraft^®^ showed slightly lower results compared to the negative control, while SMARTBONE shows slightly higher results (Figure 4A). Thereby, maxgraft^®^ exhibited significant differences compared to the negative control (*p* ≤ 0.013) as well as to the SMARTBONE^®^ material (*p* ≤ 0.008).

In the XTT assay, both test groups exhibited slightly higher results compared to the negative control, with highest values for maxgraft^®^ (Figure 4B). Thereby, the values for maxgraft^®^ were significantly different compared to the negative control (*p* ≤ 0.02), while the values for SMARTBONE^®^ did not show any significant differences to this control group (Figure 4B).

In the LDH assay, both test groups marginally exceeded the toxic limit, but showed no significant differences to the negative control (Figure 4C). Thereby, the standard variances of the maxgraft^®^ material were higher than for the SMARTBONE^®^ material (maxgraft: 44%, SMARTBONE: 1.7%). 

In all assays, the values of the positive control materials were highly significant different to the other test samples (*p* ≤ 0.02 or lower).

## 4. Discussion

The purification of the precursor tissue of natural biomaterials is of particular importance to prevent adverse clinical events such as graft rejection due to immunogenic interactions with components such as cells or transmission of pathogens [4,9,10,11]. In this context, the different available bone blocks are produced on basis of varying purification processes with different purification efforts [7]. Although all materials have been proven to fulfil the provisions of the respective norms, it has been shown that the purification results are widely differing [7]. In this context, a study of Ghanaati and Barbeck et al. including five commercially available allo- or xenogeneic bone blocks has been conducted that focused on their ultrastructure and the detection of cellular or organic matrix components based on easily applicable histological methods [7]. Interestingly, the results showed that three out of the five bone blocks contained cells or cell remnants, which led to the conclusion that these materials differ in the desired material composition originally targeted by the manufacturer and which is mandatory by different regulations and laws. In this study, the investigated test samples were divided into 4 groups according to the ultrastructures of bone matrix, remnants of collagen and cells. The classification can be distinguished as follows: complete purification of the bone matrix with a loss of the lamellar structure and without the presence of collagen as well as conservation of all previously mentioned structures.

In the present study, two other bone blocks were comparably analyzed, that is, the allogeneic block maxgraft^®^ and the xenogeneic block SMARTBONE^®^, using the same histological methods. Furthermore, cytocompatibility and physical characteristics were determined using different extract assays and SEM. Altogether, the microscopic results show that both bone blocks exhibit a trabecular structure with a lamellar suborganization. No cells or cellular remnants were found nor associated with the calcified bone matrix nor with the intertrabecular collagen matrix. Thus, both bone blocks and their underlying purification processes does not seem to differ in view of the successful removal of immunogenic components. However, the microscopic analyses revealed that the purification of both bone blocks differed with regard to their collagen contents. High amounts of the intertrabecular collagenous matrix were observable in case of the allogeneic bone blocks, while only some loosely distributed collagen remnants were found at the surfaces of the xenogeneic bone blocks. Additionally, the SEM analysis showed that island-like areas that seemed to originate from the added polymer were observable in case of the xenogeneic SMARTBONE^®^ block, while the maxgraft^®^ bone block exhibited a fibrillar surface texture.

Furthermore, a few fragments of the bone matrix were observable within the trabecular interspaces in case of the allogeneic bone block, while within the intertrabecular interspaces of the xenogeneic bone block, high numbers of matrix fragments were observed. 

Finally, the in vitro cytocompatibility analyses showed that both materials were fully cytocompatible, although the values in the LDH assay slightly exceeded the toxic limit. In this context, it should be mentioned that the LDH assay exhibits high sensitivity and the results should be compared with both the XTT and BrdU assays, which was already described by Jung et al. [8]. Thereby, since both materials showed favorable cytocompatibility in the XTT and BrdU assays, in connection with the slightly increased values in the LDH assays, an overall sufficient cytocompatibility can be assumed. 

Based on these new results both bone blocks are comparable to the bone blocks of group 2 from the previously conducted study, which means that the level of purification as well as the preservation of the bone matrix is similar to the DIZG Human Spongiosa block [6]. However, the purification level of both bone blocks seems to be more favorable as not only was the “intact” calcified bone matrix detected but also tissue-specific collagen, especially in case of the maxgraft^®^ blocks. Thus, the former classification of bone blocks must be revised and three new groups regarding the quality standards need be added (Table 1).

Altogether, the presented results show that the analyzed bone blocks have the highest level of purification, as both the calcified matrix and bone tissue-specific collagen were found without any signs of remaining cells or cell remnants.

However, the contained collagen in case of the maxgraft^®^ bone block is of special interest for the (bone) regeneration process as it has been revealed in a broad variety of studies that this molecule enables enhancement of the cell activity of bone cells and other related cell types, such as endothelial cells, and thus osteogenesis [5]. In general, collagen is necessary for wound healing and thus it is conceivable that both analyzed bone blocks may optimally support the regeneration of bone tissue even in dentistry but also in other applications such as orthopedics or traumatology [5]. However, this did not result in higher values in the extract cytocompatibility assays since L929 cells were used and were not directly seeded onto the materials. Furthermore, the composition of the extracts was not determined whether collagen or other native materials have been extracted. In this context, it should be noted that the analyzed maxgraft^®^ block is produced by the Cells + Tissuebank Austria (C+TBA) that also provides bone blocks or bone substitutes for these latter applications. Interestingly, it has been revealed in different studies that these biomaterials allow for successful bone tissue repair [15,17]. However, further studies that include molecular biological methods should be conducted to analyze the impact of the described results on the process of bone tissue regeneration.

A further interesting result was found in the presence of high numbers of matrix fragments in case of the SMARTBONE^®^ block. In this context, it was also shown that such matrix fragments, which were also found in case of the xenogeneic bone substitute material Bio-Oss^®^, can induce a fast and high implant bed vascularization, which might support the regeneration process [18]. Interestingly, these fragments may induce multinucleated giant cells that have also been detected associated to different other biomaterials and bone substitutes [9,10,18,19,20]. Moreover, it has been revealed that these cells seem to be of the foreign body giant cell phenotype, and they express both pro- and anti-inflammatory molecules [21]. Additionally, it has been shown that this cell type expresses the vascular endothelial growth factor (VEGF), which is a key element of the angiogenesis process within a tissue and a promoting factor of osteoblast differentiation and proliferation [22]. Thus, it is conceivable that the application of the SMARTBONE^®^ block might result in satisfying clinical results by supporting the bone regeneration process on its molecular level. 

Altogether, the analysis of the purification effort of both bone blocks showed that it is possible to combine the purification from cells and other immunogenic components with preservation of the bone matrix ultrastructure and the bone tissue-specific collagen. Further studies should analyze the biological activity of the included collagen and the bone matrix fragments and its consequences for the bone healing process. Furthermore, the present study results revealed that SEM investigations and cytocompatibility measurements should also be included in the preclinical evaluation of the biocompatibility of bone blocks and should be complemented by direct cell assays and the determination of extract composition.

## 5. Conclusions

In the present study, two further bone blocks of both allogeneic and xenogeneic origin were microscopically analyzed with special focus on their composition and ultrastructure, as well as on possible cell or tissue remnants. The analysis showed that both bone blocks were completely free of cellular remnants and only some collagen-like structures as well as remnants of the polymer of the SMARTBONE^®^ block were found. Both materials were cytocompatible according EN ISO 10993-5:2009. Overall, the level of purification of the maxgraft^®^ block appears to be favorable because only “intact” calcified bone matrix and higher amounts tissue-specific collagen could be detected.

## Figures and Tables

**Table 1:** Revised classification of available natural blocks for bone regeneration.

Group/Class	Lamellar Structure	Tissue-Specific Collagen	Organic/Cell Remnants in the Trabeculae	Organic/Cell Remnants on Trabeculae	CYTOCOMPATIBILITY	Bone Substitute Material
1	-	-	-	-	(?)	Bio-Oss^®^ Spongiosa
2	+	+	-	-	+	maxgraft^®^, SMARTBONE^®^
3	+	-	-	-	(?)	DIZG Human Spongiosa
4	+	-	+	-	(?)	Tutobone^®^
5	+	-	+	+	(?)	Puros^®^ Allograft Spongiosa, OsteoBiol^®^ Sp
